# The multifaceted biology of lncR-Meg3 in cardio-cerebrovascular diseases

**DOI:** 10.3389/fgene.2023.1132884

**Published:** 2023-03-10

**Authors:** Jing Li, Wenxiu Liu, Fu Peng, Xiaoyu Cao, Xiaofang Xie, Cheng Peng

**Affiliations:** ^1^ Key Laboratory of Southwestern Chinese Medicine Resources, Key Laboratory of standardization of Chinese herbal medicine of MOE, Chengdu University of Traditional Chinese Medicine, Chengdu, China; ^2^ Department of Pharmacology, Key Laboratory of Drug-Targeting and Drug Delivery System of the Education Ministry, Sichuan Engineering Laboratory for Plant-Sourced Drug and Sichuan Research Center for Drug Precision Industrial Technology, West China School of Pharmacy, Sichuan University, Chengdu, China

**Keywords:** LncR-Meg3, mechanisms, clinical application, cardiovascular diseases, cerebrovascular diseases

## Abstract

Cardio-cerebrovascular disease, related to high mortality and morbidity worldwide, is a type of cardiovascular or cerebrovascular dysfunction involved in various processes. Therefore, it is imperative to conduct additional research into the pathogenesis and new therapeutic targets of cardiovascular and cerebrovascular disorders. Long non-coding RNAs (lncRNAs) have multiple functions and are involved in nearly all cellular biological processes, including translation, transcription, signal transduction, and cell cycle control. LncR-Meg3 is one of them and is becoming increasingly popular. By binding proteins or directly or competitively binding miRNAs, LncR-Meg3 is involved in apoptosis, inflammation, oxidative stress, endoplasmic reticulum stress, epithelial-mesenchymal transition, and other processes. Recent research has shown that LncR-Meg3 is associated with acute myocardial infarction and can be used to diagnose this condition. This article examines the current state of knowledge regarding the expression and regulatory function of LncR-Meg3 in relation to cardiovascular and cerebrovascular diseases. The abnormal expression of LncR-Meg3 can influence neuronal cell death, inflammation, apoptosis, smooth muscle cell proliferation, etc., thereby aggravating or promoting the disease. In addition, we review the bioactive components that target lncR-Meg3 and propose some potential delivery vectors. A comprehensive and in-depth analysis of LncR-Meg3’s role in cardiovascular disease suggests that targeting LncR-Meg3 may be an alternative therapy in the near future, providing new options for slowing the progression of cardiovascular disease.

## 1 Introduction

Epigenetics is the study of heritable changes in gene expression that do not involve DNA sequence variation and vary over an organism’s lifetime. Mechanisms of epigenetics include DNA methylation (and demethylation), histone modifications, and non-coding RNAs such as microRNAs. On the basis of epigenetic research, the clinical implications of molecular outcomes and their potential long-lasting epigenetic bases have become increasingly clear (Zhang et al., 2020b).

Long non-coding RNAs (lncRNAs), a subset of ncRNAs, consist of RNA transcripts longer than 200 nucleotides and are incapable of being translated into proteins. Through genomics research, many lncRNAs have been found thus far. Despite the fact that no open reading frames have been identified in lncRNAs, biological investigations are increasing exponentially. Accumulating evidence suggests that lncRNAs are tightly associated with numerous cellular functions, including genomic imprinting, cell-cycle regulation, chromatin remodeling, proliferation, differentiation, senescence, apoptosis, division, and metabolism (Bridges et al., 2021). LncRNA-mediated gene expression is exquisitely involved in transcriptional regulation and RNA-protein or protein-protein formation (Peng et al., 2017). These findings would not only provide an essential hint for cellular mechanisms but also foretell an imminent outlook.

Cardio-cerebrovascular diseases are a collection of disorders of the brain and cardiovascular system, such as heart failure, atherosclerosis, stroke, cardiomyopathy, etc. It is estimated that by 2030, more than 77 million people will perish from cardio-cerebrovascular diseases, placing a massive economic burden on every nation (Béjot et al., 2016). Hence, fresh therapeutic measures for its early prevention must be developed.

LncRNAs exhibit excellent susceptibility to particular diseases, thus allowing for a high probability of profiling patients’ conditions. Moreover, the underlying mechanisms of these lncRNAs also suggest exciting opportunities for precision medicine. According to current research, numerous lncRNAs are associated with heart failure and vascular dysfunctions. H19 ameliorates myocardial infarction-induced damage and maladaptive cardiac remodeling, for instance (Zhang et al., 2020a). CHAST is an independent predictor of cardiac contractile function in patients with acute myocardial infarction (Wang et al., 2020b). Mhrt, a newly identified cardioprotective lncRNA, reflects the risk and prognosis of chronic heart failure (Zhang et al., 2019b). The therapeutic target of lncRNAs has initiated a pragmatic approach for cardio-cerebrovascular treatment (Winkle et al., 2021). Among lncRNAs related to cardio-cerebrovascular diseases, lncR Meg3 confers a high risk of cardio-cerebrovascular diseases. Therefore, in this review, we will outline not only the pathophysiology but also the precise design of agents that exhibit good potential to treat cardio-cerebrovascular diseases. We conducted a search of the current literature using keywords including lncR Meg3, heart failure, cardiomyopathy, myocardial infarction, cerebrovascular diseases, ischemic stroke, ischemia-reperfusion, atherosclerosis, congenital heart disease, and biological functions in PubMed, Web of Science, Springer, and Elsevier ScienceDirect in the latest 10 years.

## 2 Identification and characteristics of MEG3 imprint gene

**FIGURE 1:**
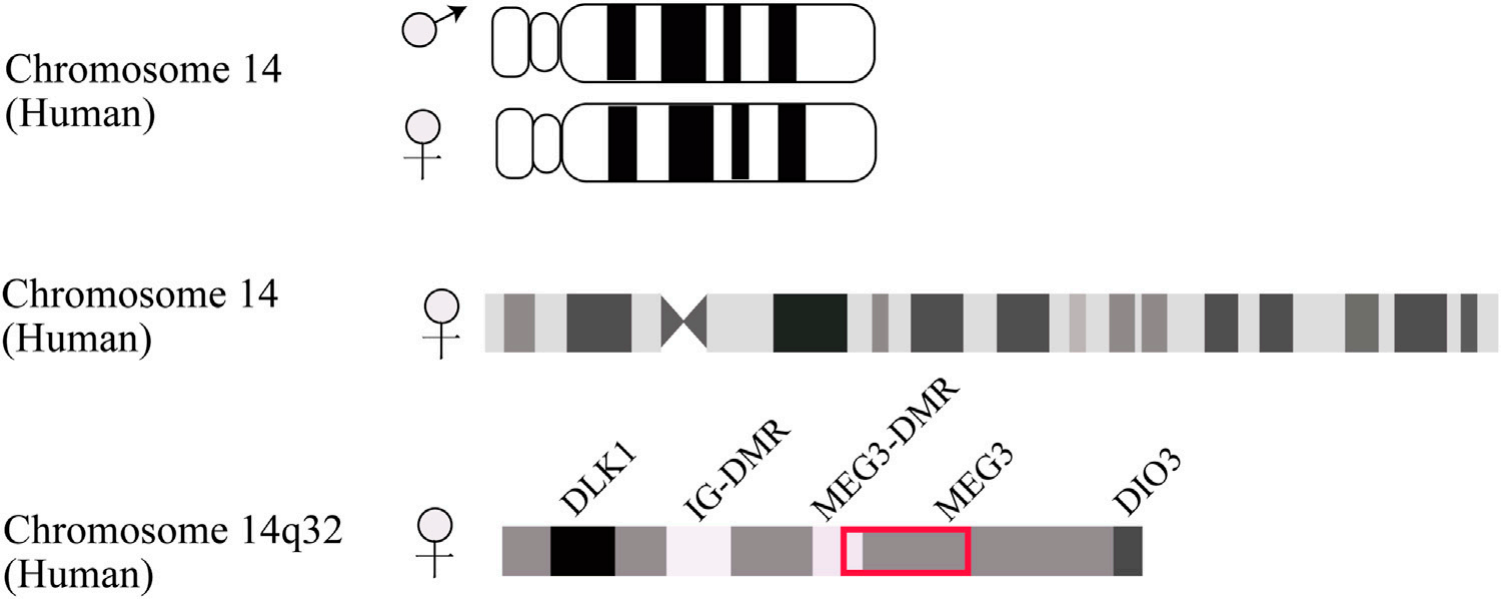
Schematic description of DLK1-MEG3 locus on human chromosome 14. The IG-DMR locates on about 13 kb upstream from MEG3 promoter. The MEG3-DMR locates on 1.5 kb upstream from MEG3 gene and overlapped with MEG3 promoter.

MEG3 is an imprinted gene initially identified as the homologue of gene trap locus 2 (Gtl2) in mice and found to serve a pleiotropic role in normal homeostatic functions (Miyoshi et al., 2000). In human beings, this maternally expressed gene mutually imprints with the paternally expressed gene delta-like homologue 1 (DLK1), forming an imprinting domain on chromosome 14q32 that contains two differentially methylated regions (DMRs), namely the intergenic DMR (IG-DMR) and post fertilization-derived secondary MEG3-DMR, as depicted in Figure 1 (Zhang et al., 2022b). MEG3 is located approximately 100 kb away from the adjacent DLK1 gene, its promoter is methylated, and it shares the same transcriptional orientation as DKL1. Indeed, MEG is deemed non-coding RNA since the undefined open reading frame lacks a Kozak consensus sequence (Zhang et al., 2010a).

Up to date, the characteristics of the MEG3 imprint gene are reflected in potential physiological or pathological activities. It has long been proposed that MEG3 acts as a significant tumor suppressor gene according to its characteristic decreased expression in some cancers, including non-functional pituitary adenoma, colorectal cancer, non-small cell lung cancer, hepatocellular carcinoma, neurospongioma, and meningeoma (Li et al., 2019a) (Lu et al., 2013). For instance, MEG3 is discovered to be abnormally elevated in type 2 diabetes patients in the clinic (Chang et al., 2020) and exacerbates insulin resistance by downregulating the miR-185-5p/Egr2 axis and upregulating Foxo1 expression (Chen et al., 2019a). Low-expressed MEG3 could upregulate miR-494-3p/OTDU4 expression and promote breast cancer growth (Zhu et al., 2022). Knockdown of MEG3 promoted hepatoma cells (SMMC-7721 and BEL-7402) by activating the PI3K/AKT pathway through regulating adaptor-related protein complex 1 (AP1G1) (Sun et al., 2019). Moreover, MEG3 can enhance the differentiation of satellite cells and govern the development of skeletal muscle (Cheng et al., 2020; Raza et al., 2020). The expression of MEG3 was detected to be declining in congenital intestinal atresia (CIA) tissues at clinic and animal levels. MEG3 increased the differentiation of bone marrow-derived stem cells (BMSCs) into intestinal ganglion cells and prevented the death of intestinal ganglion cells under hypoxia exposure to protect against CIA injury by directly modulating the miR-211-5p/GDNF axis (Xia et al., 2019). These findings highlighted the critical role of DLK1/MEG3 harmony in disease progression and overall prognosis. Our understanding of this imprint gene is still in its infancy, despite substantial development over the previous few decades. 2002 marked the first publication of the pioneering research between MEG3 and cardiovascular disorders, however, it did not highlight the boosting effects of MEG3 on heart failure (Sutton et al., 2002).

## 3 Mechanisms of lncR-Meg3

**FIGURE 2:**
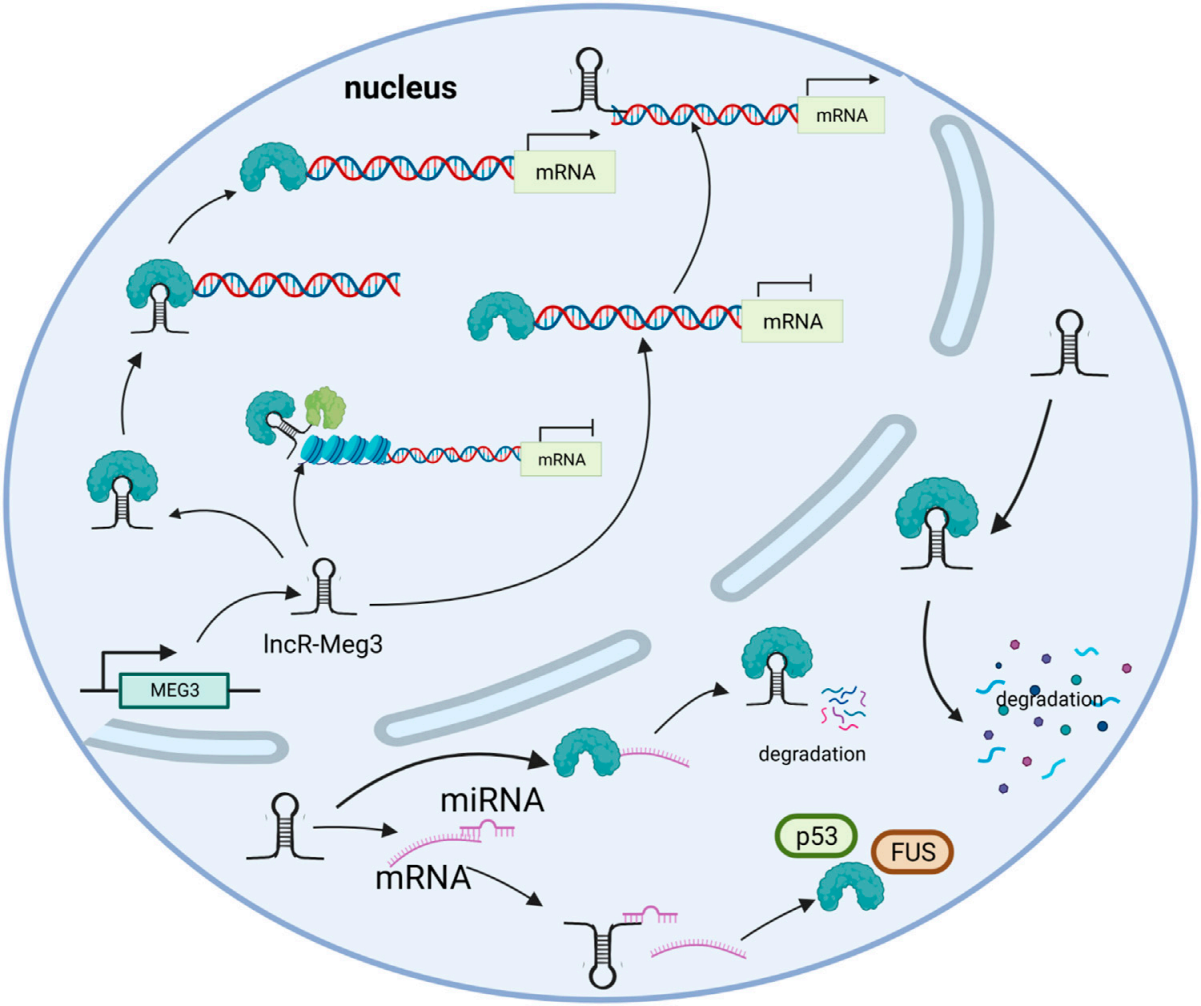
The mechanisms of lncR- Meg3. lncR- Meg3 acts as a molecular scaffold connecting different proteins and forming large complexes that regulate chromatin structure and gene expression. LncR- Meg3 competes to bind miRNAs and affects mRNA translation. LncR- Meg3 binds proteins, thereby promotes mRNA degradation and/or regulates gene expression.

LncR-Meg3 is a typical intergenic lncRNA identified in the MEG3 region, as previously stated (Zhang et al., 2010b). Numerous studies have shown that the active mechanisms of lncRNAs mostly depend on their specific targets. In addition to miRNAs, other mechanisms such as histone modification and gene expression regulation are also involved, as depicted in Figure 2.

### 3.1 Epigenetic regulation

Epigenetics is prevalent in nature, as acquired and inherited epigenetic modifications to gene expression can occur without a DNA sequence mutation (Zhang et al., 2020b). This refers to DNA methylation, histone acetylation, and miRNA regulation in general. The genomic imprinting gene, which is associated with a system that discriminates between two alleles based on their expression levels, may serve to identify parental alleles and ensure their transcriptional development (Barlow and Bartolomei, 2014). As a typical imprinted gene, the MEG3 differentially methylated region was analyzed by Erin et al. MEG3 is biallelically expressed in embryonic stem cells, while on embryonic day 12.5, MEG3 is maternally expressed. Further studies demonstrated that activating histone modifications were specific to the maternal DMR, resulting in distinct expression levels of allelic genes (McMurray and Schmidt, 2012). This phenomenon can be gleaned from diverse disease progressions, as histone modification is mainly mediated by polycomb repressive complexes (PRC). LncR-Meg3 could obviously raise the level of H3K27me3 by inducing PRC2 recruitment and modifying the type I transforming growth factor beta receptor (TGFBR1) promoter through distal regulation. Additionally, a long-range interaction between H3K4me1/MEG3 peaks and the TGFBR1 promoter revealed that MEG3/PCR2 could also regulate the activity of distal regulatory elements (Mondal et al., 2015). An in-depth study further corroborated this correlation through SHAPE-based foot printing, and significant protection at C204, C205, and C206 in the presence of PRC2 was observed in Sherpa’s study, indicating their role in genes’ assembly (Sherpa et al., 2018). JARID2, a required component of PRC2, was found to be associated with lncRNA through a 30-amino acid region. In the absence of MEG3 expression in human induced pluripotent cells, the chromatin distribution of JARID2, PCR2, and H3K27me3 is altered, suggesting a role in PRC2 recruitment (Kaneko et al., 2014). Notable is the fact that lncR-Meg3 contacts both JARID2 and EZH2, promoting their interaction and boosting the likelihood of PRC2 moving towards the target chromatins. Moreover, lncR-Meg3 dramatically upregulates the trimethylation level of H3K27 (lysine 27 on histone H3) *via* boosting EZH2 recruitment and presumably affects DLK1in a cis-repressive manner (Zhao et al., 2010).

### 3.2 Transcriptional regulation

In eukaryotes, a discontinuous transcription process is initiated by different transcription factors (TF) (Mazzocca et al., 2021). LncRNAs are believed to bind and regulate transcriptional coactivator or corepressor complexes in their capacity as protein scaffolds (Kurokawa et al., 2009). A small amount of MEG3 RNA has been shown to boost p53-mediated reporter gene expression (Wu et al., 2018). P53-dependent cell cycle genes were revealed to be regulated by p53-p21-DREAM-E2F/CHR (p53-DREAM pathway) and DREAM is a transcriptional repressor that binds to the E2F or CHR promoter (Engeland, 2018). What’s more, once the p53-DREAM pathway was disrupted, a variety of genes repressed by p53 would be overexpressed. LncR-Meg3 may promote DREAM-mediated suppression of p53-dependent genes. LncR-Meg3 may serve as a transcriptional coactivation factor that regulates the expression of proapoptotic genes. LncR-Meg3 exerts its pro-apoptotic effects in conjunction with fused in sarcoma (FUS) proteins *via* FUS/tumor lysis syndrome (TLS) transcription factors (Wu et al., 2018). In addition, a binding site for zinc-finger protein CCCTC-binding factor (CTCF) can be found in the second intron of MEG3, indicating a potential opportunity to mediate interactions (Rosa et al., 2005).

### 3.3 Post-transcriptional regulation

In general, mRNA results from DNA transcription and can be translated into proteins. Currently, mRNA treatments are broadly applied in protein replacement therapy, cancer immunotherapy, and genomic engineering (Kauffman et al., 2016). LncR-Meg3 could exacerbate ischemia-reperfusion injury, for example, *via* binding to Krüppel-like factor 4 (KLF4) (Li et al., 2020). Moreover, lncRNAs can alleviate the suppression effect on target mRNAs by acting as miRNA sponges. By binding competitively with miRNA, lncR-Meg3 enables the downstream target gene suppressors of cytokine signaling 6 (SOCS6) to reduce the suppression impact on miR-19b, thereby inhibiting HG-induced apoptosis through the JAK2/STAT3 signaling pathway (Xiao et al., 2020). High levels of lncR-Meg3 expression in atherosclerosis can bind to miR-361-5p to modulate ABCA1 expression and induce cell apoptosis (Wang et al., 2019). This mechanism is ubiquitous in cardio-cerebrovascular diseases and provides a therapy strategy. LncRNAs have the potential to interact with proteins and influence gene expression.

## 4 LncR-Meg3 and cardio-cerebrovascular diseases

### 4.1 LncR-Meg3 and cardiovascular diseases

LncR-Meg3 displays an indispensable role in various cardiovascular diseases, including heart failure, cardiomyopathy, and myocardial infarction (Table 1).

**TABLE 1:** Roles of lncR-Meg3 in different cardiovascular diseases.

Disease	Subjects	Change	Signaling pathway	Literature
Heart failure	Human: umbilical vessels and cord bloods	Down-regulated	Modulating epigenetic regulation	Yu et al. (2019)
Heart failure	Mice	Up-regulated	Serving as a substrate for lncRNA AK045171	Xu et al. (2020)
Heart failure	Mice	Up-regulated	Inhibiting Mmp-2 promoter	Piccoli et al. (2017)
Heart failure	Mice, Cardiomyocytes	Up-regulated	Regulating miR-361-5p/HDAC9 axis inducing cardiac hypertrophy	Zhang et al. (2019a)
Heart failure	Cardiac fibroblasts	Up-regulated	Regulating JAK2, STAT3 signaling pathways, regulating the balance of proliferation and apoptosis	Li et al. (2021)
Myocardial infarction	Human: plasma	Up-regulated	Serving as a biomarker for myocardial infarction	Wei and Wang (2021)
Myocardial infarction	Mice, Primary neonatal mice ventricular myocytes	Up-regulated	Regulating NF-κB- and ERS-associated apoptosis, serving as a p53 protein target	Li et al. (2019b)
Myocardial infarction	Rat& mice&human:Cardiomyocytes	Up-regulated	Regulating p53 and induce Meg3-FUS formation and promoting cardiomyocyte apoptosis	Wu et al. (2018)
Myocardial infarction	H9C2 cells	Up-regulated	Inducing hypoxia-induced injury by modulating miR-325-3p/TRPV4 axis	Zhou et al. (2021)
Myocardial infarction	H9C2 cells	Up-regulated	Regulating cell’s apoptosis	Yang et al. (2019)
Diabetic cardiomyopathy	Rat: cardiomyocytes	Up-regulated	Promoting mitochondria-mediate apoptosis pathway, influencing mitochondria-mediate apoptosis pathway	Zhang et al. (2019c)
Ventricular septal defect	Human: all heart tissues and blood samples, rat	Down-regulated	Regulating miR-7-5p/EGFR axis and enhancing autophagy	Cao et al. (2019)
Viral myocarditis	Mice, Macrophages	Up-regulated	Sponging for miR-485, inhibiting inflammation and promote M2 macrophage polarization	Xue et al. (2020)
Congenital heart disease	Human: blood samples	Down-regulated	Serving as a possible biomarker for congenital heart disease	Chang et al. (2021)

#### 4.1.1 LncR-Meg3 and heart failure

Heart failure is a heterogeneous syndrome that occurs in the terminal stages of various heart diseases. Increasing workload causes cardiomyocyte enlargement in adult hearts, and this process can be controlled in pathological situations (Nakamura and Sadoshima, 2018). MEG3 has been proven to be enriched in TAC cardiac fibroblasts (Uchida, 2017).

Following transcription, MEG3 remodifies protein function by interacting directly with the target protein or its binding partners. In a cell model of angiotensin-induced hypertrophy, MEG3 was observed to upregulate histone deacetylase 9 (HDAC9) by competitively binding with miR-361-5p (Zhang et al., 2019a). Signal transducer and activator of transcription 3 (STAT3) has been linked to a number of pathological processes in heart failure, including ECM accumulation, collagen generation, and inflammatory responses, all of which mediate MEG3 activation in various cardiovascular signal transduction pathways. During myocardial remodeling, MEG3 also regulated the TGF-β signaling pathway. Mondal’s work demonstrated an RNA-DNA triplex structure over MEG3 binding sites associated with TGF-β pathway genes, indicating a tight relationship between MEG3 and TGF-β. Subsequent research revealed that when TGF-β was present, lncR-Meg3 was overexpressed, causing cell apoptosis (Mondal et al., 2015). Through binding to TGF-β, the methylation of lncR-Meg3 varied dynamically and aggravated myocardial fibrosis (Zhang et al., 2018b). P53 is a transcription factor that influences the development of the cell cycle and apoptosis. Initial studies verified that lncR-Meg3 could enhance p53 function by acting on target genes including C-reactive protein (CRP), intercellular cell adhesion molecule-1 (ICAM-1), vascular endothelial growth factor (VEGF), and hypoxia inducible factor-1 (HIF-1) (Song et al., 2019). Matrix metalloprotease 2 (MMP-2) is a typical TGF-β1-induced product that can be regulated directly by combining with p53. MMP-2 and its active cleaved form were both suppressed in TAC mice, resulting in cardiac hypertrophy, fibrosis, and apoptosis. As proved in Piccoli’s study, inhibition of MEG3 impeded MMP-2 production, hence preventing cardiac fibrosis and diastolic dysfunction (Piccoli et al., 2017).

Moreover, lncR-Meg3 can serve as a substrate and be regulated by other lncRNAs. By endogenously combining with SP1, lncR-AK045171 was capable of elevating the transcriptional activation of MEG3, resulting in alleviating heart failure (Xu et al., 2020). Chronic pulmonary hypertension can cause right heart failure and ultimately death. In the pulmonary artery smooth muscle cells (PASMCs), lncR-Meg3 interacted with and inhibited expression of miR-328-3p, which then led to the upregulation of insulin-like growth factor 1 (IGF1R) and ultimately promoted cardiomyocyte hypertrophy, according to the recent study (Xing et al., 2019).

#### 4.1.2 LncR-Meg3 and cardiomyopathy

Cardiomyopathy refers to a category of disorders characterized by heart muscle dysfunction. Cardiomyopathies are classified into two types: primary and secondary cardiomyopathies (Brieler et al., 2017). Primary cardiomyopathy is produced mostly by hereditary factors and is categorized into three categories based on etiology and pathology: dilated cardiomyopathy, hypertrophic cardiomyopathy, and hypertrophic cardiomyopathy, which can lead to exertional dyspnea, heart failure, and sudden cardiac death (Mao et al., 2021). Secondary cardiomyopathy, often known as “specific cardiomyopathy”, refers to heart muscle abnormalities produced by established causes or occurring after other disorders. Mounting evidence has associated MEG3 with a large range of cardiomyopathies, and it is worth exploring their molecular mechanisms (Japp et al., 2016).

There are no specific treatments for virus infection, which is the primary cause of viral myocarditis (VMC). It is a type of secondary cardiomyopathy that is detrimental to human health and is a miscellaneous disease that needs to be treated in the near future. By endogenous binding to miR-223 and targeting TNF receptor related factor 6 (TRAF6), down-regulated MEG3 increases body weight, survival, left ventricular ejection fraction (LVEF), and left ventricular fraction shortening (LVFS) in VMC mice. This can be achieved without activating the NF-κB signaling pathway (Xue et al., 2020).

Diabetic cardiomyopathy is a cardiac complication occurring in the late stages of diabetes mellitus. Distinct from the primary types, diabetic cardiomyopathy often appears in the absence of coronary artery disease, hypertension, and valvular heart disease (Dillmann, 2019). Diabetic cardiomyopathy is closely related to MEG3. Depletion of MEG3 in a hyperglycemic cell model exacerbated inflammatory injury and activated the TGF-signaling pathway by increasing TGF-1, SMAD2, and SMAD7 expression (Wang et al., 2018). In detail, the ratio of Bax/Bcl-2 is self-regulated in healthy bodies. Nevertheless, a high glucose situation breaks this balance. Several studies have verified that lncR-Meg3 is upregulated in patients with diabetes mellitus and in the mouse model of streptozotocin administration (Zhang et al., 2019c). Cell apoptosis is commonly observed in high glucose conditions, and this process is mainly mediated by mitochondrial metabolism. Upregulation of MEG3 facilitates apoptosis by promoting mitochondria-mediated intrinsic apoptosis pathway. An in-depth investigation revealed that the lncR-Meg3/miR145/PDCD4 axis was engaged in diabetic cardiomyopathy, showing chaos in MEG3 expression and hastening heart failure (Chen et al., 2019b). Moreover, analysis of 53 peripheral blood samples from individuals with type 2 diabetes mellitus (T2DM) showed that MEG3 is related to the progression of T2DM and can be used as a novel biomarker for clinical diagnosis (Chang et al., 2020).

Also, ferroptosis is a type of cell death that is caused by the buildup of iron-dependent lipid peroxides, which can happen when there is persistent hyperglycemia (Xu et al., 2019). It was shown that marker events of ferroptosis occur with concomitant enhanced production of lncR-Meg3 after oxygen and glucose deprivation. The lethality of elastin and RAS-selective lethal 3 (RSL3), essential factors of ferroptosis such as ferritin heavy chain 1 (FTH1), acyl-CoA synthetase long chain family member 4 (ACSL-4), and GPX4 (glutathione peroxidase 4), were significantly increased. Treatment with si-Meg3 can turn this situation around (Chen et al., 2021).

#### 4.1.3 LncR-Meg3 and myocardial infarction

Myocardial infarction (MI) caused by post-acute or persistent hypoxia is a prevalent disease in the 21st century. Different types of collagens replace the primary tissues during the process, resulting in irreversible heart failure. It's critical to stop this disease in its tracks, and lncR-Meg3 fulfils this purpose well (Gao et al., 2021b).

MI is characterized by inflammation and myocardial apoptosis, which can eventually lead to myocardial dysfunction and heart failure (Marchant et al., 2012). The expression of lncR-Meg3 was increased in ischaemic tissues and hypoxic neonatal mouse ventricular myocytes (NMVMs). Through the p53 pathway, overexpression of lncR-MEG3 may induce NF-κB and ERS-mediated apoptosis. Knockdown of lncR-MEG3 protects cardiomyocytes from hypoxia-induced apoptosis and ROS induction, thereby reducing cardiac remodeling and enhancing cardiac function (Li et al., 2019a). In addition, under hypoxic conditions, Meg3 is directly up-regulated by p53 and is involved in apoptosis regulation *via* direct binding to the RNA-binding protein FUS (Wu et al., 2018). After MI, the P53-induced Meg3-FUS complex plays an important role in the death of myocardial cells. In Zhao’s work, lncR-Meg3 was found to induce apoptosis by increasing the expression of FoxO1 and damaging the myocardial cells under hypoxia-ischemic conditions (Zhao et al., 2019).

In hypoxic conditions, transient receptor potential cation channel subfamily V member 4 (TRPV4) expression was significantly increased by competitively binding with miR-325-3p, whereas the effect was reduced by downregulation of lncR-Meg3, indicating a negative feedback loop between lncR-Meg3 and cell survival (Zhou et al., 2021). Knockdown of MEG3 alleviates hypoxia-induced H9c2 cell injury by miR-183-mediated suppression of p27 through activation of PI3K/AKT/FOXO3a signaling pathway (Gong et al., 2018). Zhang et al. discovered a circulating MEG3/miR-223 axis in recent years, which was further corroborated by sequence complementarity with base pairs that lncR-Meg3 was capable of impeding the function of miR-223 and thus enhancing cell pyroptosis (Zhang et al., 2018a).

In a previous narration, lncR-Meg3 was found to recruit PRC through the EZH2 subunit and catalyze the methylation of histones in order to exert epigenetic regulation. Yet, another chromosome binding protein, high mobility group box1 (HMGB1) also regulates lncR-Meg3. In contrast to methylation modification, HMGB1 expression was altered by means of ceRNA, with miR-22 serving as an intermediary between lncR-Meg3 and HMGB1 (Fluri et al., 2015). Furthermore, small nucleolar RNAs (snoRNAs) can be utilized as an innovative approach to evaluate the risk of MI. When zooming in on different genes in 14q32 locus, 53 single nucleotide polymorphisms in MEG3 were included, eight of which were associated with cardiovascular endpoints. Based on this adjuvant therapy, linkage between MEG3, snoRNAs, and MI should be established as early as possible (Håkansson et al., 2019).

LncR-Meg3 was determined to be involved in MI, although more attention was focused on apoptosis. It is undeniable that angiogenesis accounts for a large portion of this ischemic recovery. Previous research has linked MEG3 enrichment in endothelial cells to angiogenesis by regulating the cell cycle, migration, proliferation, and differentiation (Gong et al., 2018) (Shen et al., 2022). Increasing the local concentration of VEGF in the infarct area can stimulate new angiogenesis in the infarct area, improve blood supply to the ischemic myocardial muscle, decrease the infarct area, and thus improve cardiac function.

**FIGURE 3:**
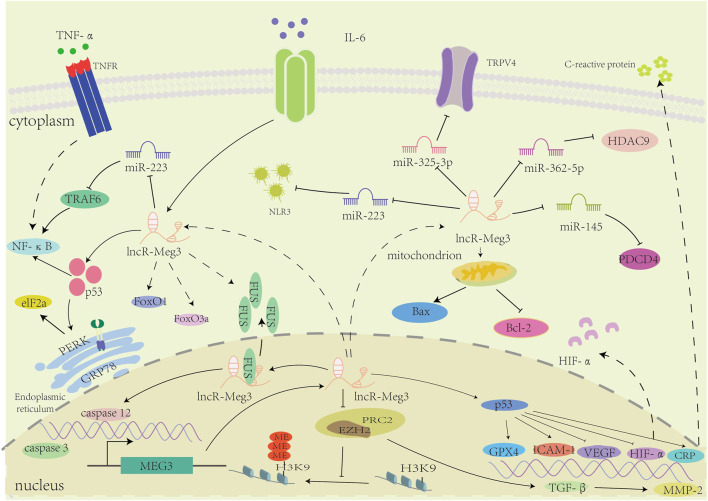
The mechanisms of involvement of lncR-Meg3 in cardiovascular diseases. LncR-Meg3 regulates cardiovascular diseases through diverse mechanisms. By directly interacting with different miRNAs, lncR-Meg3 regulates the expression of target genes or proteins, facilitating the progression of diseases. In nucleus, the PRC2 subunit EZH2 mediates the methylation of H3 thereby regulating the gene’s expression.

In general, lncR-Meg3 plays a regulatory role in a variety of cardiovascular diseases, as depicted in Figure 3.

### 4.2 LncR-Meg3 and cerebrovascular diseases

Cerebrovascular diseases encompass a wide range of vasculovascular dysfunctions in the brain, including ischemic stroke, ischemia-reperfusion, and atherosclerosis. LncR-Meg3 plays a crucial role in Cerebrovascular diseases and is currently a hot research topic (Table 2).

**TABLE 2:** LncR-Meg3 roles in cerebrovascular diseases.

Disease	Subjects	Change	Signaling pathway	Literature
Ischemic stroke	Human: serum	Up-regulated	Regulating cell’s proliferation and apoptosis	Liu et al. (2021)
Ischemic stroke	Human: blood	Up-regulated	An independent risk factor in ischemic stroke	Han et al. (2018)
Ischemic stroke	Human: blood, HUVECs	Up-regulated	An independent risk factor in ischemic stroke	Du et al. (2021)
Ischemic stroke	Mice, HBMECs	Up-regulated	Independent prognostic marker	Wang et al. (2020a)
Ischemic stroke	HUVECs	Up-regulated	Stimulating endothelial sprouting angiogenesis	Ruan et al. (2018)
Ischemic stroke	Rat	Up-regulated	Promoting angiogenesis by activating Notch signaling	Liu et al. (2017)
Ischemia-reperfusion	Rat Neurocytes	Up-regulated	Sponging for miR-485, promoting pyroptosis and inflammation	Liang et al. (2020)
Ischemia-reperfusion	BV2 cells	Up-regulated	Regulating microglial polarization	Li et al. (2020)
Ischemia-reperfusion	HT22 cells	Up-regulated	Regulating autophagy through miR-181c-5p/ATG7 signaling pathway	Li et al. (2022)
Ischemia–reperfusion	SD rats, H9C2 cells, 293 cells	Up-regulated	Regulating miR-7-5p/PARP1 axis inducing I/R injury	Zou et al. (2019)
Ischemia–reperfusion	RBMVECs	Up-regulated	Modulating p53/GPX4 axis, influencing cell’s ferroptosis	Chen et al. (2021)
Atherosclerosis	Human: coronary artery disease tissues	Up-regulated	Sponging for miR-21, regulating endothelial cell proliferation and migration	Wu et al. (2017)
Atherosclerosis	Human: VSMCs & coronary artery disease tissues	Down-regulated	Sponging for miR-26a, modulating the balance of proliferation/apoptosis	Bai et al. (2019)
Atherosclerosis	mice	Up-regulated	Regulating cell proliferation and impairing capillary-like formation	Bai et al. (2019)
Atherosclerosis	VSMCs	Up-regulated	Regulating miR-361-5p/ABCA1 axis, promoting VSMCs proliferation and inhibiting apoptosis	Wang et al. (2019)
Atherosclerosis	VSMCs	Down-regulated	Targeting p53	Liu et al. (2019)
Atherosclerosis	VSMCs	Down-regulated	Inhibiting miR-125a-5p, increasing IRF1 expression	Zheng et al. (2019)
Cerebral infarction	RBMVECs	Up-regulated	Regulating angiogenesis after cerebral infarction through p53/NOX4 axis	Zhan et al. (2017)

#### 4.2.1 LncR-Meg3 and ischemic stroke

Cerebral ischemic stroke is one of the leading causes of morbidity and mortality worldwide and is rapidly increasing annually with no more effective therapeutic options. This disease occurs due to a sudden blockage of arteries (ischemic stroke) or an aberrant blood flow into brain tissue when a blood vessel ruptures (hemorrhagic stroke), resulting in neurological dysfunction, cognitive deficits, and even dementia by reducing oxygen and glucose levels (Paul and Candelario-Jalil, 2021). The main therapies for ischemic stroke are thrombolytic methods with endovascular thrombectomy or recombinant tissue plasminogen activator. Because a major disadvantage of these approaches may occur during treatment, an increasing number of studies are beginning to focus on new pharmaceutical therapies (Sun et al., 2018). Recently, lncRNAs have been proposed as new targets for modulating the pathological process of ischemic stroke.

In ischemia-induced stroke (IS), abnormal expression of lncR-Meg3 plays a critical role in brain injury. In a mouse model of middle cerebral artery occlusion (MCAO), the high MEG3 group had a shorter survival time than the low MEG3 group. The expression pattern profiled an IS onset, a significant increase in lncR-Meg3 was observed in the first 4 h and remained stable within 48 h, indicating that 4 h post-ischemic is the prime time for treatment and that measures should be taken to prevent this catastrophic disease as soon as possible (Wang et al., 2020a).

In the pathological course of an IS, brain tissue reacts to hypoperfusion by getting less blood and oxygen and turning on the expression of apoptosis-related factors. This causes cells to die because they can’t get enough energy. It has been mentioned that oxygen-glucose deprivation (OGD) treatment would elevate the expression of MEG3, Bax, and cleaved caspase-3 in human brain microvascular endothelial cells and cause a high apoptosis rate. Furthermore, by acting on miR-21, lncR-Meg3 suppresses the oxidative stress response, preventing the secretion of TNF-α, IL-6, and IL-17A (Liu et al., 2021). P53 is also activated by MEG3 and functions as a transcriptional regulator in DNA-damaged cell death. MEG3 binds directly to the p282 DNA-binding domain (DBD) containing amino acids 53-271 (p282-DBd53-271) in order to stimulate p53-mediated trans-activation and mediate ischemic neuronal death (Yan et al., 2016). Determination of the expression levels of lncR-Meg3 in peripheral blood mononuclear cells would aid in the prediction of IS risk, as the alteration of inflammatory cells is an important index in IS etiopathogenesis.

LncR-Meg3 has also been identified as a crucial regulator in neuronal cell death, typically *via* ceRNA, which functions as a miRNA during hypoxia or ischemic disease. Han et al. found that lncR-Meg3 can protect against ischemic damage and improve neurobehavioral outcomes by taking down the expression of miR-21 (Yan et al., 2017), miR-181b (Han et al., 2018), miR-485 (Liang et al., 2020), and miR-378 (Luo et al., 2020). What’s more, lncR-Meg3 also functions in hypoxic-ischemic brain damage. Silencing of MEG3 or upregulation of miR-129-5p can effectively ameliorate inflammatory symptoms by cooperating with dexmedetomidine in neonatal C57/BL6 mice, thereby preventing the animals from acute mortality and chronic nervous system injury (Zhou et al., 2018).

By reconstructing or enhancing cerebral blood flow in ischemic areas, also known as angiogenesis, vascular remodeling has recently been regarded as a crucial method for treating IS (Seto et al., 2016). The P53 pathway was involved in lncR-Meg3 induced vascular remodeling. Sun found that inhibition of lncR-Meg3 promoted more smooth muscle cells’ growth from G0/G1 phase to the G2/M + S phase, and p53 engagement was observed in experiments (Sun et al., 2017). In a three-dimensional angiogenesis model, the formation of vessel-like structures was enhanced under constitutive stimulation of the Notch signaling pathway, whereas blocking this signaling pathway, partially inhibited network formation (Liu et al., 2003). During ischemic brain injury, lncR-Meg3 downregulation increased angiogenesis and reduced cerebral lesions, mediated by the Notch signaling pathway. Further research demonstrated that MEG3 negatively regulated the Notch pathway both *in vitro* and *in vivo* (Liu et al., 2017). MEG3 is a critical regulator of angiogenesis following ischemic brain injury. In addition, neural stem cell (NSC) proliferation is the initial phase of neuronal regeneration and has been implicated in physiological and pathological processes following IS. MEG3 inhibits NSC proliferation following IS by upregulating miR-493-5p and possibly downregulating MIF (Zhao et al., 2021). This provides a new potential direction for targeted therapy for IS.

In conclusion, MEG3 plays a crucial role in accelerating neuronal cell death under conditions of cerebral ischemia or hypoxia, leading to damage and dysfunction of brain tissue.

#### 4.2.2 LncR-Meg3 and ischemia-reperfusion

Cerebral ischemia-reperfusion is a complicated injury characterized by high rates of mortality and morbidity worldwide. A substantial variety of mechanisms, such as apoptosis, inflammatory and oxidative reactions, are involved, and lncR-Meg3 has been reported to regulate the adverse symptoms by serving as a sponge for miRNAs.

During cerebral I/R, the MEG3/miR-485/AIM2 axis contributes to pyroptosis by activating caspase-1 signaling (Liang et al., 2020). MCAO induces pyroptosis and the release of IL-1 and IL-18. Overexpression of MEG3 increases the expressions of AIM2, ASC, cleaved caspase, and GSDMD-N, and promotes caspase-1 signaling. In the absence of melanoma 2 (AIM2) inflammasomes, the adaptor protein apoptosis speck-like protein (ASC) is recruited and activates caspase-1. Knockdown of MEG3 would inhibit these.

It has been shown that severe cerebral ischemia/reperfusion injury induces high levels of autophagy and neuronal death. In HT22 cells treated with oxygen and glucose deprivation/reoxygenation (OGD/R), MEG3 expression was significantly upregulated, and autophagy was increased, whereas knockdown of MEG3 expression greatly reduced autophagy. Furthermore, MEG3 binds to and suppresses the expression of miR-181c-5p, whereas miR-181c-Sp binds to and suppresses the expression of the autophagy-related gene ATG7 (Li et al., 2022). By activating the Wnt/β-catenin signaling pathway, down-regulation of MEG3 expression can improve nerve growth after cerebral IRI in rats, reduce brain infarct size, and alleviate nerve damage in MACO rats (You and You, 2019). Moreover, MEG3 inhibition reduces brain I/R injury by inhibiting M1 polarization and promoting M4 polarization *via* KR Uppel-like factor 4 (KLF4) (Li et al., 2020). These findings provide a solid theoretical foundation for potential therapeutic targets of brain I/R injury.

#### 4.2.3 LncR-Meg3 and atherosclerosis

Atherosclerosis (AS) is a chronic disease that can induce lesions and cause various complications. Endothelial dysfunction and leukocyte infiltration into the endothelium precede atherosclerotic formation, which is followed by fatty streaks, intermediate and advanced lesions, and fragile plaques. Chronic inflammation, vascular smooth muscle cells’ (VSMCs) phenotypic switching, and neovascularization are all crucial processes in the progression of atherosclerotic lesions and plaque rupture.

The transition of VSMC phenotype from a systolic to a proliferative state is an important factor in atherosclerosis, angiogenesis, and neointima formation. The activation of lncR-Meg3 inhibited VSMC proliferation and promoted cell apoptosis. However, increasing miR-26a levels can counteract the effect of lncR-Meg3 *via* the SMAD signaling pathway, alleviating atherosclerosis symptoms (Bai et al., 2019). By coordinating with miR-21, lncR-Meg3 weakened the expression of cyclin D1, ki-67, and proliferating cell nuclear antigen (PCNA), thereby accelerating the apoptosis of endothelial cells and inhibiting cell proliferation and migration (Wu et al., 2017). A decrease in type Ⅰ and type Ⅳ collagen can also be observed in patients with atherosclerosis, hinting at potential role of lncR-Meg3 in fibrosis (Wu et al., 2017). Furthermore, by targeting miR-361-5p, miR-204 (Yan et al., 2019), and miR-26a (Bai et al., 2019) to regulate ABCA1 and CDKN2A, MEG3 inhibition can promote smooth muscle cell proliferation, inhibit apoptosis, and reduce inflammation. This evidence suggests that MEG3 could be used as a biomarker and therapeutic strategy to reduce and reverse atherosclerosis.

**FIGURE 4:**
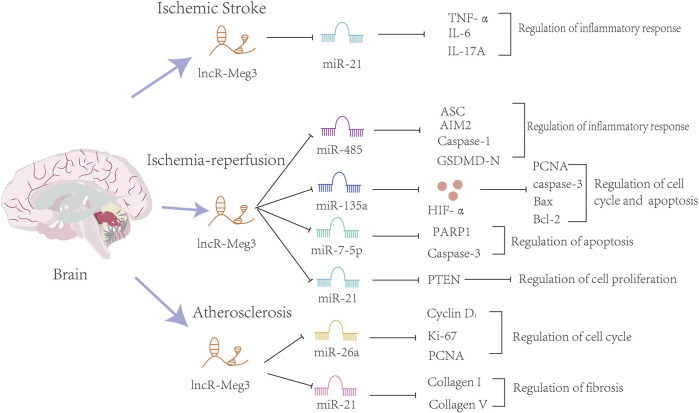
The mechanisms of the involvement of lncR-Meg3 in cerebrovascular diseases. Several miRNAs such as miR-21, miR-482, miR-135a, miR-7-5p, miR21, miR-26a and miR-21 play a role in these diseases. These miRNAs inhibit the expression of downstream target genes. Serving as a sponge, lncR-Meg3 degrades miRNAs and influences the progression of inflammatory response, cell cycle and apoptosis.

Overall, lncR-Meg3 exerts indispensable roles in cerebrovascular diseases, and the whole mechanism is summarized in Figure 4.

### 4.3 Others

The interaction of genetic abnormalities and environmental factors is what causes congenital heart disease (CHD). Imprinted genes regulated by epigenetic modifications are essential for normal embryonic development. Diverse selected genes such as MYH7, GATA4, NKX2-5, and TBX5 are involved in this phenotypic spectrum, and they may undergo modifications from lncRNAs (Baban et al., 2022). Taking some examples, in ventricular septal defects (VSD), lncR-Meg3 was found to be directly targeted by miR-7-5p and significantly inhibited autophagy through EGFR signaling pathway (Cao et al., 2019). Six imprinted genes, including MEG3, were downregulated in CHD children compared to healthy individuals, according to a study of 27 children with CHD. Risk analysis shows that a specific methylation level range presages the occurrence of CHD and can be utilized as a novel biomarker for efficacious diagnosis (Chang et al., 2021).

Atrial fibrillation (AF), the ultimate stage of numerous heart disorders, carries a 22%–26% lifetime risk. RNA-sequencing experiments have identified that thousands of ncRNAs are involved in AF, and the vast majority of them are located in the syntenic region of the DLK1-DIO3 locus, sharing a location similar to that of MEG3 (Leblanc et al., 2021).

## 5 Clinical applications

### 5.1 LncR-Meg3 and diagnosis

Conventional diagnostic approaches include patient history inquiries, physical examinations, blood pressure measurements, *etc.* The purpose of these technologies is to determine whether there is coronary artery narrowing or abnormal protein levels in plasma or lesion tissues. However, some limitations of these diagnostic methods, such as their low sensitivity and short detection time, delay the optimal treatment time for patients with MI. Hence, there is an urgent need for novel biomarkers with high sensitivity and specificity. Owing to its long half-life, lncRNA has become an optimal candidate for health evaluation (Lu et al., 2015).

The correlation between lncR-Meg3 and acute myocardial infarction was confirmed (Wei and Wang, 2021). The researchers measured the serum concentrations of dimethylglycine (DMG), Apelin-12, and lncR-Meg3 in 110 patients with acute chest pain for more than 6 h, Results showed that the levels of lncR-Meg3 in acute myocardial infarction (AMI) groups were approximately two times higher than those in healthy individuals. However, there were no statistically significant differences among DMG, lncR-Meg3, and Apelin-12. The receiver operating characteristic (ROC) curve uncovered the potential value of lncR-Meg3 as a new biomarker for predicting the occurrence of MI. Using 0.015 as the critical value for MEG3-mRNA, the specificity and sensitivity are 81.58% and 85.29%, respectively, which is marginally superior to DMG (71.05%). Additionally, a panel of factors indicated that except for lncR-Meg3, the proportion of men, history of myocardial infarction, smoking, and cognitive heart failure in the AMI group can also serve as independent risks for MI. Compared with traditional biomarkers, such as cardiac troponin I and creatine phosphokinase-isoenzyme-MB, these new markers possess higher sensitivity and a longer detection window (Wei and Wang, 2021).

Sepsis is a serious systemic inflammation that can lead to life-threatening organ failure and ultimately cause long-term morbidity. Analysis of plasma samples from 82 patients showed that the expression levels of lncR-Meg3 were tightly correlated with sepsis mortality, and lower levels of lncR-Meg3 can be found in the survival group instead of the mortality group (Chen et al., 2019a).

Moreover, lncR-Meg3 alteration is associated with coronary artery disease (CAD), and Bai collected 40 abnormal tissues and 35 normal coronary arteries. Using RT-PCR, it was determined that lncR-Meg3 was lower in CAD patients than in healthy individuals (Bai et al., 2019).

Among various epigenetic mechanisms, the potential association between aberrant DNA methylation and CHD is becoming increasingly apparent. DNA methylation is highly dynamic with the character of demethylation during cardiomyocyte development. A pilot study on congenital heart diseases analyzed this disease based on DNA methylation (Chang et al., 2021). Stratified analysis showed that the methylation of gDMR of eight imprinted genes was altered, including GRB6, MEST, PEG10, NAP1L5, INPP5F, PLAGL1, NESP, and MEG3. Various degrees of methylated imprint genes depend on different types, but they share a common descending tendency in CAD tissues. Compared to individuals, the level of methylation of MEG3 decreased from 45.31 to 39.53 percent, which was associated with an increased risk of coronary heart disease.

In conclusion, MEG3 is expected to be used in the diagnosis of some cardiovascular diseases, but it still needs to be identified with a large sample size, and the relationship between genes and diseases still needs further experimental studies.

### 5.2 lncR-Meg3 and treatments

Due to their great sensitivity and specificity, lncRNAs are considered possibilities for innovative therapeutic uses at the present time. Some approaches such as RNA technologies, antisense oligonucleotides, and small molecule inhibitors, have been utilized for curative treatments. Moreover, some bioactive compounds and medicines have been explored for their potential roles in lncRNA regulation, but their potential needs to be further demonstrated. The following is a list of possible drugs that target lncR-Meg3 (Table 3).

**TABLE 3:** Potential LncR-Meg3-targeting compounds.

Subject	Formula	Model	Change	Reference
Inorganic arsenic	**—**	A549 cells	Up-regulated	Wang et al. (2021a)
IL-10		Receptor activator of nuclear factor-B ligand-induced osteoclast differentiation model	Down-regulated	Gao et al. (2022)
Methylene blue	C_16_H_18_ClN_3_S	Rabbit model of osteoarthritis	Up-regulated	Li et al. (2018)
Protocatechuic aldehyde	C_7_H_6_O_3_	PC12 cell injury model induced by hydrogen peroxide	Down-regulated	Zhong et al. (2020)
Selenium	—	HCY-induced fibrosis in cardiac fibroblasts in mouse	Down-regulated	Li et al. (2021)
Arsenic trioxide	—	Hepatocellular carcinoma cells	Up-regulated	Fan et al. (2019)
Melatonin	C_13_N_2_H_16_O_2_	Rats model of febrile convulsion	Down-regulated	Wu et al. (2021)
Schisandrin A	C_24_H_32_O_6_	choriocarcinoma JEG-3 and BeWo cells	Up-regulated	Ji and Ma (2020)
orexin-A	C_152_H_243_N_47_O_44_S_4_	Rats model of central precocious puberty	Down-regulated	Tao et al. (2015)
Fucoidan	(C_14_H_21_NO_11_) n	Fibrotic buccal submucous fibroblasts	Down-regulated	Fang et al. (2022)
Fenofibrate	C_20_H_21_ClO_4_	Pancreatic cancer cell	Up-regulated	Hu et al. (2016)
Atorvastatin	C_33_H_35_FN_2_O_5_	Hypoxia cardiac progenitor cell (CPC) model	Down-regulated	Su et al. (2018)
High-content hydrogen water	—	Non-alcoholic fatty liver disease mice model and cellular model	Up-regulated	Wang and Wang (2018)
Pioglitazone	C_19_H_20_N_2_O_3_S	Subjects with MetS	Up-regulated	Liu et al. (2016)
Marsdenia tenacissima extract	**—**	Glioma cells	Up-regulated	Chen et al. (2023)
Paclitaxel	C_47_H_51_NO_14_	non-small cell lung cancer cells	Up-regulated	Xu et al. (2018b)
Metformin	C_4_HN_5_	Rats model of polycystic ovary syndrome	Up-regulated	Liu et al. (2022)
Propofol	C_12_H_18_O	Inflammatory model of LPS-stimulated rats astrocytes	Down-regulated	Zhang et al. (2022a)
Lidocaine	C_14_H_22_N_2_O	Hela cervical cancer cells	Up-regulated	Zhu and Han (2019)
Curcumin	C_21_H_20_O_6_	Gemcitabine-resistant non-small cell lung cancer cells	Up-regulated	Gao et al. (2021a)
N-acetylcysteine	C_5_H_9_NO_3_S	Rats model of liver cirrhosis	Down-regulated	Mohamed et al. (2020)

Protocatechuic aldehyde (PA), a bioactive compound extracted from *S. miltiorrhiza*, possess anti-oxidative and anti-inflammatory activities. Zhong et al. found that damage in H_2_O_2_-stimulated PC12 cells was ameliorated by PA management. More importantly, PA decreased apoptosis-associated factors levels in H_2_O_2_-triggered PC12 cells were also reversed by MEG3 overexpression. Conclusively, they promulgated the activation of Wnt/β-catenin and PTEN/PI3K/AKT pathways were accelerated by PA *via* suppressing MEG3, which offered a reference for clinical research of PA for the treatment of Spinal cord injury (SCI) (Zhong et al., 2020). Baicalin, a bioactive compound derived from Scutellaria baicalensis Georgi, obtained greater property to develop novel therapeutic approaches for CVDs. Liu et al. discovered that knockdown of endogenous MEG3 promoted proliferation and migration and inhibited apoptosis in HA-VSMCs, while Baicalin reversed these effects (Liu et al., 2019). Furthermore, lncR-Meg3 induced p53 expression and blocked AMPK activation through lncR-Meg3/p53/AMPK signaling pathways, inspiring us to excavate therapy at the gene level (Yang et al., 2019). Cardiac fibrosis is a common characteristic that can also be observed in hyperhomocysteinemia. It’s conceivable that retarding the speed of fiber formation would alleviate aberrant remodeling in patients with hyperhomocysteinemia. Selenium (Se), an essential mineral crucial for cardiovascular health, has attracted considerable attention in recent years. Li et al. discovered that MEG3 played an important role in Se stimulation. In accordance with the impact of Se, silencing MEG3 decreased the expression levels of -SMA, collagen I, and collagen III, consequently slowing the rate of fibrosis (Li et al., 2021).

## 6 Knowledge gap and future directions

In a nutshell, although more and more researchers are curious about this “junk” production, a paucity of mechanisms for lncR-Meg3 in cardio-cerebrovascular diseases still needs to be further elucidated. Overcoming these obstacles is significant for developing more precise drugs for different diseases as early as possible. Herein, we systematically summarize some controversies and limitations that remain unresolved, hoping to provide novel warrants for further investigation.

LncRNAs have been verified to possess many biological functions and regulatory roles. They can influence gene transcription *via* chromatin modification and participate in the majority of biological processes. LncRNAs could regulate gene expression by interacting with RNAs and/or proteins. After blinding with lncRNAs, RNAs and proteins would also be affected. More importantly, lncRNAs can function as ceRNAs that bind to target miRNAs like a sponge. Evidence supports that lncR-Meg3 interacts with miRNA directly or competitively in tumors, metabolic diseases, immune system diseases, and cardio-cerebrovascular diseases, the mechanisms of which are tightly associated with apoptosis, proliferation, inflammation, and oxidative stress. In gastric cancer cell (AGS) models, overexpression of MEG3 inhibited epithelial-mesenchymal transition (EMT) by decreasing MMP-3 and MMP-9 levels and thereby inhibiting cell migration (Xu et al., 2018a). lncR-Meg3 can also enhance the apoptosis of hypoxic cardiomyocytes *via* activating FOXO1 signaling pathway (Zhao et al., 2019). A variety of regulatory roles of lncR-Meg3 are found in cardiomyocytes, fibroblasts, and endothelial cells, which may hamper the study of their molecular mechanisms for cardio-cerebrovascular diseases. Similarly, we recognized that the consistency of lncRNA as a therapeutic target is also uncertain, and it may vary in different cardiovascular and cerebrovascular diseases mentioned above, such as MEG3, which was up-regulated in Ang-II-treated cardiomyocytes (heart failure) while down-regulated in congenital heart disease. Is MEG3 only applicable to some specific therapeutic targets of cardio-cerebrovascular diseases? A larger cohort study is needed. For another, a majority of studies of the etiology of MEG3 and cardio-cerebrovascular diseases rely on experiments in immortalized and primary cells and lack relevant *in vitro* data analysis. Direct homologues of MEG3 have been identified in mice, and subsequent studies, including various models to challenge MEG3 knockout mice or overexpression, such as TAC surgery, are mandatory.

The chromosomal region 14q32 contains several imprinted genes, which are expressed either from the paternal (DLK1 and RTL1) or maternal (MEG3, RTL1as, and MEG8) allele only (Zhang et al., 2022b). In the nucleus, MEG3 regulates adjacent or distal gene expression in a cis-or trans-regulated manner. The regulation of cis-genes by lncRNAs is determined not only by the one-to-one effect of lncRNAs on neighboring genes, but also part of a complex regulatory unit, in which the expression of a protein-coding gene may be regulated by two or more lncRNAs and the coregulation between transcriptional dependent and transcriptional independent. Given that MEG3 interferes with nearby genes and silencing of MEG8 impairs endothelial function *via* increasing tissue factor pathway inhibitor 2 (TFPI2), an inhibitor of angiogenesis. DLK1 plays an inhibitory role in cardiac fibroblast-to-myofibroblast differentiation by interfering with TGF/Smad-3 signal pathway in the myocardium (Rodriguez et al., 2019). So, it is essential to investigate whether MEG3-adjacent gene expression contributed to its regulation of endothelial cells and myocardial fibrosis.

Antisense oligonucleotides (ASOs) or small interfering RNAs (siRNA) could target lncRNAs and down regulate levels, for being stable and easy entry into cells (Zhao et al., 2023). Meanwhile, for cerebrovascular diseases, lncRNA drugs need to break through the permeability and brain targeted delivery system of the blood-brain barrier (BBB). Microcarriers and exosomes can assist in the transmission of information between cancer cells and between cancer cells and adjacent cells. In Yu’s study, a pegylated cationic liposome (RGD Lip) modified with arginine-glycyl-aspartic peptide (RGD) as a novel gene delivery system was designed (Yu et al., 2018). The results showed H19x siRNA was efficiently transferred into the placenta of C57BL/6 mice. Wang designed a novel polymerized nanoparticle that targets lncRNA INK4 as well as T cell immune receptors with Ig and ITIM domains (TIGIT)/poliovirus receptors (PVR) to inhibit liver cancer (Wang et al., 2021b). Exosomes harboring amounts of lncR-Meg3 can serve as a convenient and non-invasive biomarker for early detection. However, there are still inadequate clinical trials, and their feasibility and safety need to be further demonstrated. Moreover, enriched with bioactive compounds, natural plants are usually included in today’s regimens to treat diverse cardio-cerebrovascular diseases (Mishra et al., 2019). Although limited studies have been performed, the combination of compounds with lncRNA will help obtain better therapeutic effects in clinical applications.

## 7 Conclusion

Above all, direct or indirect evidence from clinical or experimental research has proven an aberrant elevation of lncR-Meg3 expression in a variety of cardio-cerebrovascular diseases, which would play a significant role in the occurrence and development of these conditions. Multiple deleterious stimulations can finally lead to the abnormal expression of MEG3, and these mechanisms are mostly related to epigenetic, transcriptional, and post-transcriptional regulation. In these cases, lncR-Meg3 mostly served as a ceRNA to modulate the degradation of miRNAs, thereby circumventing their inhibitory effect on target mRNAs. It is worth noting that the mechanisms have not been fully elucidated, and more efforts in this field, both in experiments and in the clinic, are required. Clinical studies have shown that CKMB and cTnI have high specificity and sensitivity in the diagnosis of AMI. However, their value in the early diagnosis of AMI was limited because the changes were significant at about 7 h after the occurrence of MI. If we could establish the characteristic relationship between lncR-Meg3 and markers of myocardial injury, it would more strongly prove that MEG3 can be used as a biomarker for the cardiovascular diseases listed. Many bioactive compounds derived from natural plants have been shown to have the ability to regulate lncRNA expression *via* molecular mechanisms, and MEG3 research may offer new strategies for finding pharmacotherapy in the treatment of cardio-cerebrovascular diseases.

## Glossary

MEG3: maternally expressed gene 3
lncRNA: long non-coding RNA
ncRNA: non-coding RNA
DMR: differentially methylated region
PRC: polycomb repressive complexes
EZH2: enhancer of zeste homolog 2
Glt2: gene trap locus 2
DLK1: delta-like homologue 1
TGFBR1: type I transforming growth factor beta receptor
H3K27: lysine 27 on histone H3
FUS: fused in sarcoma
TLS: tumour lysis syndrome
CTCF: CCCTC-binding factor
KLF4: Krüppel-like factor 4
SOCS6: suppressors of cytokine signaling 6
PDCD4: programmed cell death 4
HDAC9: histone deacetylase 9
EMC: endoplasmic reticulum membrane protein complex
MMP-2: matrix metallopeptidase 2
TAC: transverse aortic constriction
ICAM-1: intercellular adhesion molecule 1
CRP: C-reactive protein
VEGF: vascular endothelial-derived growth factor
PASMCs: pulmonary arterial smooth muscle cells
IGF1R: insulin like growth factor 1 receptor
VMC: Viral myocarditis
TRAF6: TNF receptor associated factor 6
LVEF: left ventricular ejection fraction
T2DM: type 2 diabetes mellitus
RSL3: RAS-selective lethal 3
FTH1: ferritin heavy chain
ACSL-4: acyl-CoA synthetase long chain family member 4
GPX4: glutathione peroxidase 4
TRPV4: transient receptor potential vanilloid 4
HMGB1: high mobility group box1
snoRNAs: small nucleolar RNAs
MCAO: middle cerebral artery occlusion
HIBD: hypoxic-ischemic brain damage
SNPs: single nucleotide polymorphisms
AIM2: absent in melanoma 2
ASC: apoptosis speck-like protein
PTEN: phosphatase and tensin homologue
PARP1: Poly(ADP-ribose) polymerase 1
VSMCs: Vascular Smooth Muscle Cells
PCNA: proliferating cell nuclear antigen
EC: endothelial cell
PDGF: platelet derived growth factor
IRF1: interferon regulatory factor
CAD: coronary artery diseases
CVD: cardiovascular disease
HUVEs: human umbilical vein endothelial cells
DNMT: DNA methyltransferase
VEGF: vascular endothelial-derived growth factor
ERK1/2: extracellular signal-regulated kinase 1/2
CHD: congenital heart disease
VSD: ventricular septal defect
OGD: oxygen and glucose deprivation
ASOs: Antisense oligonucleotides
siRNA: small interfering Rnas
TFPI2: tissue factor pathway inhibitor 2
ARP1: adaptor-related protein complex 1
CIA: congenital intestinal atresia
TLS: tumor lysis syndrome
STAT3: Signal transducer and activator of transcription 3
